# Tumor‐Microenvironment‐Responsive Nanomedicine for Enhanced Cancer Immunotherapy

**DOI:** 10.1002/advs.202103836

**Published:** 2021-11-19

**Authors:** Shaojun Peng, Fengfeng Xiao, Meiwan Chen, Huile Gao

**Affiliations:** ^1^ Zhuhai Institute of Translational Medicine Zhuhai Precision Medical Center Zhuhai People's Hospital (Zhuhai Hospital Affiliated with Jinan University) Zhuhai Guangdong 519000 China; ^2^ State Key Laboratory of Quality Research in Chinese Medicine Institute of Chinese Medical Sciences University of Macau Macau 999078 China; ^3^ Key Laboratory of Drug‐Targeting and Drug Delivery System of the Education Ministry and Sichuan Province Sichuan Engineering Laboratory for Plant‐Sourced Drug and Sichuan Research Center for Drug Precision Industrial Technology West China School of Pharmacy Sichuan University Chengdu 610041 China

**Keywords:** drug delivery, immunotherapy, nanomedicine, stimulus‐responsive, tumor microenvironment

## Abstract

The past decades have witnessed great progress in cancer immunotherapy, which has profoundly revolutionized oncology, whereas low patient response rates and potential immune‐related adverse events remain major clinical challenges. With the advantages of controlled delivery and modular flexibility, cancer nanomedicine has offered opportunities to strengthen antitumor immune responses and to sensitize tumor to immunotherapy. Furthermore, tumor‐microenvironment (TME)‐responsive nanomedicine has been demonstrated to achieve specific and localized amplification of the immune response in tumor tissue in a safe and effective manner, increasing patient response rates to immunotherapy and reducing the immune‐related side effects simultaneously. Here, the recent progress of TME‐responsive nanomedicine for cancer immunotherapy is summarized, which responds to the signals in the TME, such as weak acidity, reductive environment, high‐level reactive oxygen species, hypoxia, overexpressed enzymes, and high‐level adenosine triphosphate. Moreover, the potential to combine nanomedicine‐based therapy and immunotherapeutic strategies to overcome each step of the cancer‐immunity cycle and to enhance antitumor effects is discussed. Finally, existing challenges and further perspectives in this rising field with the hope for improved development of clinical applications are discussed.

## Graphical abstract

Recent progress of tumor‐microenvironment‐responsive nanomedicine for cancer immunotherapy, which responds to the signals of weak acidity, reductive environment, high‐level reactive oxygen species, hypoxia, overexpressed enzymes, and high‐level adenosine triphosphate is summarized.

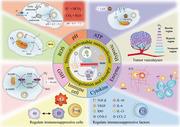

## 1 Introduction

Cancer remains one of the leading diseases that threaten the health of humans, leading to 9.96 million deaths worldwide in 2020.^[^ ^1^ ^]^ Although substantial efforts have been devoted to conquer cancer, the achievements made thus far are still unsatisfactory, with limited improvement in five year survival rates of the patients.^[^ ^2^ ^]^ Currently, traditional tumor treatment methods include surgery, radiotherapy, and chemotherapy, which still exhibit unsatisfactory clinical benefits and severe side effects. Therefore, there is desperate need to explore and develop new cancer treatment modalities with high efficiency and few side effects.^[^ ^3^ ^]^ In 1893, William Coley, an orthopedic surgeon in America, accidentally found that suppurative streptococcal infection after surgery gave rise to tumor regression in patients with sarcoma, which opened the preface of tumor immunotherapy.^[^ ^4^ ^]^ After a century of development, cancer immunotherapy has revolutionized oncology and provided new treatment options for many types of cancer that are difficult to treat with common methods.^[^ ^5^ ^]^ In 2011, the US food and drug administration (FDA) approved the first immune checkpoint inhibitor (anti‐CTLA‐4 monoclonal antibody, Ipilimumab) for the treatment of advanced melanoma, which marked a new era of tumor immunotherapy.^[^ ^6^ ^]^ Due to the remarkable success of immunotherapy in clinical cancer treatment, the 2018 Nobel Prize in Physiology and Medicine has been awarded to James P. Allison and Tasuku Honjo for their pioneering work in discovering immune checkpoints.

Generally, the purpose of cancer immunotherapy is to train host immune cells in lymphoid tissue and antitumor immune cells in the tumor microenvironment (TME) to identify and destroy tumor cells.^[^ ^7^ ^]^ Furthermore, antitumor immune response initiated by immunotherapy could promote systemic immune monitoring, leading to the elimination of local and distant metastasis.^[^ ^8^ ^]^ In addition, immunotherapy could build up long‐term immune memory, regulate immune protection, and prevent tumor recurrence.^[^ ^9^ ^]^ Despite outstanding achievements accompanied by therapeutic efficacy, there are still clinical failures and obstacles in cancer immunotherapy.^[^ ^10^ ^]^ For instance, the delivery kinetics are limited, and the overall response rate from immunotherapy remains relatively low (usually 10–30% response rates, depending on the type of cancer) because tumor develops multiple resistance pathways, the molecular, cellular, and TME levels.^[^ ^11^ ^]^ Unlike traditional cancer treatments, regulating antitumor immunity demands accurate activation of a complex immunological system at multiple levels.^[^ ^12^ ^]^ Therefore, it is necessary to achieve precise control over immune cells in both intracellular and extracellular sites in a spatiotemporal manner, which is, however, difficult to achieve.

The most recent three decades have witnessed the great success of nanomedicines in tumor therapy.^[^ ^13^ ^]^ Since the first liposomal doxorubicin (Doxil) was approved by the US FDA for the treatment of ovarian cancer, dozens of nanomedicines have been approved for the clinical treatment of various cancers worldwide.^[^ ^14^ ^]^ Nanomedicines exhibit the advantage of preferentially accumulating in solid tumors due to the abnormally leaky vasculature and dysfunctional lymphatic drainage within the TME, which are well‐known as the enhanced permeability and retention (EPR) effect.^[^ ^15^ ^]^ Numerous studies have shown that nanomedicines exhibit the advantages of controllable drug delivery and modular flexibility, which provide an opportunity for immunotherapy to promote clinical transformation in a safe and effective manner.^[^ ^16^ ^]^ For example, a nanomedicine‐based drug delivery system could reduce off‐target toxicity and immune‐related adverse events, which are particularly significant for potent immunotherapies that could cause severe dose‐limiting toxicity, such as the cytokine storm.^[^ ^17^ ^]^ In addition, nanomedicine could target immune cells, such as effector T cells, regulatory T cells, dendritic cells, natural killer (NK) cells, and tumor‐associated macrophages (TAM), which significantly enhance their antitumor activity.^[^ ^14^, ^18^ ^]^ Moreover, nanomedicine‐based drug delivery system could further improve the pharmacological properties of the loaded immunomodulator, and protect biological drugs from premature release or degradation in vivo.^[^ ^13^, ^19^ ^]^ Furthermore, various drug delivery systems with adjustable physiochemical properties (e.g., size, shape, and surface parameters) or multiple functions can facilitate inhibitory or stimulatory actions to the immune system which exert synergistic effects for combined cancer immunotherapy.^[^ ^20^ ^]^ To achieve the multifunctional ability of nanomedicines in immunotherapy, stimuli‐responsive units were usually designed and incorporated into the nanoparticles, which respond to internal stimuli, such as pH, redox potential, hypoxia, enzymes, and adenosine triphosphate (ATP) in the TME or external stimuli such as light, ultrasonic waves, X‐rays, and electrical and magnetic fields.^[^ ^21^ ^]^ Compared with external stimuli, TME‐responsive nanomedicine exhibits the advantage that it is safe and convenient to implement without the need of outside equipment, which has attracted increasing attention in cancer immunotherapy.^[^ ^22^ ^]^

**Scheme 1:**
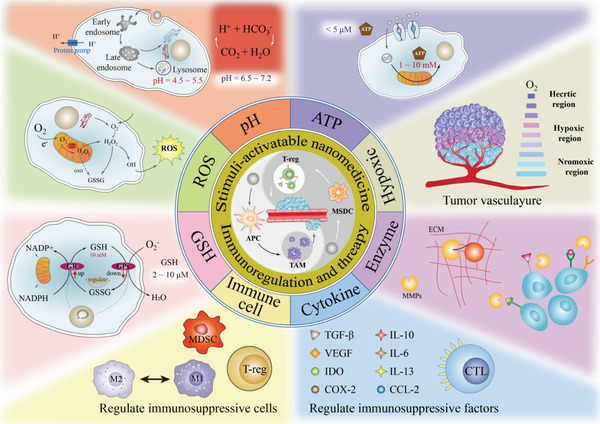
Schematic diagram of TME‐responsive nanomedicine for cancer immunotherapy.

In this review, we summarize the latest research progress on TME‐responsive nanomedicine for antitumor immunotherapy, as shown in **Table** **1**. Particular focus is given to molecular and nanoengineering approaches which could achieve the controlled functionality and immune effects of TME‐responsive nanomedicine for cancer immunotherapy (**Scheme** **1**). Such immunotherapeutic nanomedicine exhibits spatiotemporal controllability and controlled immune activation in response to TME signals, which show great potential for ameliorating several limitations and shortcomings of traditional immunotherapies to facilitate the better translation of cancer immunotherapy.

**Table 1:** Summary of TME‐responsive nanomedicine for cancer immunotherapy

Delivery platform	Immunotherapeutic drug	Sensitive molecules/bond	Source
pH‐responsive nanomedicine			
Galactosyl dextran‐retinal (GDR) nanogels	OVA	Hydrazine bond	[23]
DEX–HAase nanoparticles	3‐(bromomethyl)‐4‐methyl‐2,5‐furandione		[24]
PCL–Hyd–PEG vesicles	Antigens HCP, adjuvants CpG ODN	PCL–Hyd–PEG	[25]
Dendrigraft poly‐l‐lysines	Zoledronic acid (ZA)	1,6‐Bis(4‐formylbenzoyloxy) hexane	[26]
RPTDH NPs	R848	Poly‐l‐histidine (PHis)	[27]
Poly(ethylene glycol)‐block‐poly(diisopropanol amino ethyl methacrylate‐co‐hydroxyethyl methacrylate) (PDPA)–PPa	Small interfering RNA (siRNA)	OEI‐C14	[28]
PCPP hybrid micelles	PD‐L1‐blockade siRNA, MTPP(PS)	Amide bond	[29]
Nanogels	PTX, IL‐2	Chitosan polymers	[30]
CaCO_3_ matrix	CpG ODNs, IDOi, Ca^2+^	CaCO_3_	[31]
H‐MnO_2_ nanoshells	Ce6, DOX	H‐MnO_2_	[32]
Poly(ethylene glycol)‐block‐poly(D,L‐lactide) (PEG‐*b*‐PLA) NPs	CDNs	cytosine (C)	[33]
		pHLIP	[34]
Hollow silica nanoparticle	Catalase, Ce6		[35]
PDPM NPs	OVA	PDPA tertiary amino groups	[36]
PD‐L1 binding peptide conjugate (DCS) NPs	DOX, D‐PPA	Maleic acid amide bond	[37]
Sensitive cluster nanoparticles (SCNs)	1. 4‐[2((1R,2R)‐2‐Hydroxycyclohexylamino)‐benzothiazol‐6‐yloxyl]‐pyridine‐2‐carboxylic acid methylamide (BLZ‐945), Pt‐based prodrug	Hydrophobic–hydrophilic transition	[38]
STING‐NPs	Cyclic guanosine monophosphate‐adenosine monophosphate (cGAMP)	Polymersomes	[39]
Mesoporous silica nanoparticles (MSNPs)	Mitogen‐activated protein kinase kinase inhibitors (MEKi)	Anionic polymer polyacrylic acid	[40]
MnO_2_ particles	IPI549	MnO_2_	[41]
Hierarchical‐responsive nanoconjugates (HRNs)	Docetaxel (DTX)	P(C7A‐*r*‐DTX) block	[42]
AuNPs	DOX, hydroxychloroquine (HCQ)	HCQ prodrug	[43]
Human serum albumin	DOX prodrug	2,3‐Dimethylmaleic amide bond	[44]
Mannose‐modified PEGylated poly(lactic‐co‐glycolic acid) (MAN‐PEG‐PLGA)	Tumor associated antigens (TAA)	Hydrazone bond	[45]
poly(l‐histidine), HA	R848, DOX	Hydrazone bond	[46]
Micelleplexes	siRNA–PD‐L1	GA, C7A, DPA, C4A	[47]
Metal‐organic frameworks (MOFs)	TAAs, CpG ODNs	Lanthanide ions and GMP	[48]
GSH‐responsive nanomedicine			
HA–CD	PPa, JQ1	Disulfide bond	[49]
Redox‐activatable liposome (RAL)	PPa, IDO inhibitor	Disulfide bond	[50]
Polysaccharide fucoidan and polyamide‐amine (PAMAM) dendrimer	Verteporfin (VP), MnO_2_ NPs	Disulfide bond	[51]
Ds‐sP NPs	PS TCPP‐TER	Disulfide bond	[52]
Biodegradable mesoporous silica nanoparticles (bMSN)	CpG ODN, Ce6	Disulfide bond	[53]
Hollow mesoporous organosilica nanoparticles (HMONs)	HCPT, siMCT‐4	Disulfide bond	[54]
Copolymer nanoparticles	SN38, DMXAA	Disulfide bond	[55]
Protein nanogel		Disulfide bond	[56]
Light‐inducible nanocargo (LINC)	PPa, NLG919, OXA	Disulfide bond	[57]
HCNSP nanovector	PPa, NLG919	Disulfide bond	[58]
polyacrylic acid (PAA)–polyethyleneimine (PEI) 600	OVA	Disulfide bond	[59]
N′‐bis(acryloyl)cystamine (BISS)	Cisplatin prodrug, IR820, DTX	Ester bond	[60]
HA nanohydrogel	Oncolytic viruses (OVs)	Disulfide bond	[61]
Human serum albumin	PTX, siPD‐L1	Disulfide bond	[62]
ROS‐responsive nanomedicine			
3s‐PLGA–PEG NPs	OVA	Peroxalate ester bond	[63]
3s‐PLGA–PO–PEG/PEI	OVA	Peroxalate ester bond	[64]
Albumin	aPD1, aCD47	bis‐N‐hydroxy succinimide modified 2,2′‐[propane‐2,2‐diylbis(thio)]diacetic acid (NHS–IE–NHS)	[65]
Polypeptide‐based gel	aPD‐L1, D‐1MT	l‐Methionine (Me) and D‐1MT	[66]
Hydrogel	aPD‐L1, GEM	TSPBA	[67]
Aspirin polymeric	4‐formylbenzeneboronic acid		[68]
T7‐PEG–N,N,N‐Trimethyl chitosan(TMC)–4‐nitrophenyl‐4‐(4,4,5,5‐tetramethyl‐1,3,2‐dioxaborolan‐2‐yl) benzyl carbonate(NBC) NPs	siRNA–PD‐L1, DOX	T7‐PEG‐TMC‐NBC	[69]
Hypoxia‐responsive nanomedicine			
Mesoporous silica	CpG ODN, Ce6	Azobenzene linker	[70]
Phthalocyanine derivative	AQ4N	AQ4N	[71]
MFNs	DOX	polypropylene oxide‐*b‐* poly(6‐(2‐nitroimidazol‐1‐yl)hexyl methacrylate) (PEO‐*b*‐PNIHM)	[72]
mPEG–poly(γ‐propargyl‐L‐ glutamate (PPLG)	DOX	*p*‐Aminobenzyl groups	[73]
Enzyme‐responsive nanomedicine			
mPEG–Pep–IDOi	IDO inhibitor, ICG	mPEG–Pep–IDOi	[74]
MSNs	DOX	Peptide substrate of MMPs	[75]
Triglycerol monostearate	DOX	Triglycerol monostearate (TGM)	[76]
Poly(ethyleneglycol)‐*b*‐poly(L‐lysine) (PLL)–1‐mt, HA–Ce6	aPD‐L1, Ce6, 1‐mt	Hyaluronic acid–Ce6	[77]
Triphenylphosphine‐modified‐poly(L‐lactic acid) (TPT polymer)	DOX, d‐LND	HA–DOX	[78]
DNA nano‐cocoons (DNCs)	aPD1, CpG ONDs	Single‐stranded DNA, TGM	[79]
IR780‐M‐APP NPs	APP	MMP‐2 cleavable peptide sequence	[80]
ATP‐responsive nanomedicine			
CpG–cApt	CpG ODN, Ca^2+^	ATP‐specific aptamer	[81]
PCL–PEI–PBA	PD‐L1 siRNA, IR780	PBA	[82]
Multiresponsive nanomedicine			
BCPN	OXA prodrug, NLG919	Disulfide bond, PEGylated OXA prodrug	[83]
PEG–2‐propionic‐3‐methylmaleic anhydride (CDM)–PEI–P(CURDT)	Curcumin, NLG919	Disulfide bonds, CDM	[84]
Nanoparticle assembled from DEAP molecule	PD‐L1 antagonist, NGL919, and a substrate peptide of MMP‐2	DEAP molecule, peptide substrate of MMP‐2	[85]
Azide–PEG–PAsp	aPD‐1, PTX	MMP‐2 sensitive peptide linker, acid‐labile bond	[86]
BRNPs	d‐SN38, d‐LND	PEGylated bilirubin, d‐SN38, d‐LND, disulfide bond	[87]

## 2 The Features of the Tumor Microenvironment

**Scheme 2:**
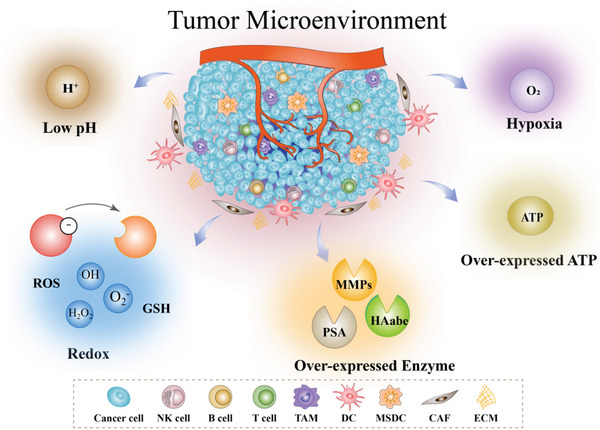
Illustration of specific biosignals in the TME, including low pH, redox, overexpressed enzymes, hypoxia, and overexpressed ATP.

Compared with normal tissues, tumors have developed unique microenvironments during their evolution.^[^ ^88^ ^]^ The TME is regarding the soil that cultivates cancer cells, and it deeply influences the occurrence and growth of tumors.^[^ ^89^ ^]^ The TME typically exhibits a more acidic microenvironment, high‐level reactive oxygen species (ROS) and glutathione (GSH), higher hypoxic status, overexpressed enzymes, and high‐level ATP, due to the fast proliferation and metabolism of tumors compared with normal tissues (**Scheme** **2**). The TME not only facilitates tumor angiogenesis and metastasis, but can also cause treatment resistance and failure.^[^ ^90^ ^]^ Therefore, the TME has become an important hallmark for the treatment of cancer and is widely used as a stimuli to control the release of drugs for immunotherapy.

### 2.1 Low pH

A large number of studies have shown that the extracellular space of the tumor tissue is weakly acidic with a pH ranging from 6.5 to 6.8 due to the deregulated energy metabolism, insufficient perfusion, and the accumulation of lactic acid, which is known as the Warburg effect.^[^ ^91^ ^]^ The elevated acidity in the tumor extracellular environment is a typical pathological feature of solid tumor tissues compared to the neutral environment of normal tissues, which has led to the development of weak acid‐activated nanomedicines or nanoprobes in recent years.^[^ ^92^ ^]^ For example, cis‐maleic monoamides were relatively stable at pH 7.4 but degraded completely at pH 6.5 for several hours, leading to the transition of surface charge.^[^ ^93^ ^]^ In addition to the mild acidity in the TME, a more significant pH decrease can be found in the intracellular lysosomal and endosomal compartments, where the pH value is between 4.5 and 6.5. It should be noted that the strong pH difference between extracellular environment and endosomes are not cancer specific since both normal cells and tumor cells exhibit acidic surroundings in endosomes. Such acidity can be utilized as a promising endogenous stimulus for the development of pH‐responsive nanomedicine. Generally, nanomedicine with tertiary amine groups exhibit a sensitive pH–stimuli‐responsive property due to the protonation of tertiary amine groups in acidic surroundings.^[^ ^94^ ^]^ Moreover, several acid‐labile linkers such as maleimide, cis‐aconityl, and hydrazones have been extensively explored for the development of pH‐responsive nanomedicine.^[^ ^95^ ^]^

### 2.2 High‐Level GSH

GSH is one of the most abundant reductive cellular metabolites, and it plays an important role maintaining the balance of the redox state in cells.^[^ ^96^ ^]^ Besides, GSH is involved in regulating protein folding by mediating the generation and degradation of disulfide bonds in many proteins.^[^ ^97^ ^]^ Generally, the concentration of GSH in tumor cells is reported to be 5 × 10^−3^–10 × 10^−3^ m, which is much higher than that in normal cells (1 × 10^−3^–5 × 10^−3^ m).^[^ ^98^ ^]^ More importantly, the concentration of GSH in the cytosol was found to be ≈1000‐fold higher than that in the extracellular environment or plasma due to the catalytic conversion of oxidized glutathione (GSSG) to GSH by GSH reductase and nicotinamide adenine dinucleotide phosphate (NADPH) in cytosol.^[^ ^99^ ^]^ Therefore, GSH has been widely applied as specific marker to achieve selective drug release in the tumor cytosol by incorporating disulfide bonds or diselenide bonds in nanomedicine.^[^ ^100^ ^]^

### 2.3 High‐Level ROS

ROS, especially hydrogen peroxide (H_2_O_2_), plays a vital role in diverse physiological processes.^[^ ^101^ ^]^ It has been demonstrated that ROS are crucial molecules that influence the occurrence and development of tumors.^[^ ^102^ ^]^ Most tumor cells produce more ROS than normal cells through pathways involving the mitochondrial respiratory chain and nicotinamide adenine dinucleotide phosphate oxidase.^[^ ^103^ ^]^ Additionally, genetic changes and changes in energy metabolism patterns in tumor cells can facilitate the production of ROS. It has been calculated that the H_2_O_2_ concentration in the TME can reach up to 100 × 10^−6^ m, which is ≈100 times higher than the level in normal tissues, which makes it a hallmark in the TME for stimuli‐responsive nanomedicine.^[^ ^104^ ^]^

### 2.4 Hypoxia

Hypoxia is a distinctive feature of most solid malignant tumors, and it plays a key role in tumor angiogenesis, metastasis, and drug resistance.^[^ ^105^ ^]^ Due to the rapid growth of cancer cells, most nutrients and oxygen are required inside the tumor, which causes vascular defects in the tumor site and the formation of irregular microvessels, resulting in damage to the microcirculation. The oxygen partial pressure gradually decreases from the tumor surface to the core. Compared with the oxygen partial pressure of 30–40 mm Hg in tumor tissue, it can be reduced to 0–2.5 mm Hg in some areas, which makes the tumor environment hypoxic.^[^ ^106^ ^]^ Hypoxia hinders the metabolic activity of cells, leading to tumor cell adaptation to hypoxia stress through a series of hypoxia‐inducible factors (HIF), primarily HIF‐1.^[^ ^107^ ^]^ This hypoxia adaptation changes the general biochemical environment around cells, and affects processes, such as cell energy metabolism,^[^ ^108^ ^]^ endocytic receptor internalization,^[^ ^109^ ^]^ transmembrane receptor recirculation, and transportation.^[^ ^110^ ^]^ Due to the significant difference between tumor tissue and normal tissue, hypoxia is evolving into the main target of diagnosis and treatment.^[^ ^111^ ^]^ Therefore, hypoxia can be used as an endogenous stimulus for tumor treatment and imaging. The functional groups that respond to hypoxia are mainly quinone, nitroaromatic, and azobenzene derivatives, which have been extensively utilized as hypoxia‐responsive nanomedicine or nanoprobe.^[^ ^111^, ^112^ ^]^

### 2.5 Overexpressed Enzymes

Enzymes, a kind of protein or RNA, are a substantial constituent of the biotechnological toolbox that provides prospective abilities and ideal characteristics to accelerate chemical reactions.^[^ ^113^ ^]^ Enzyme‐catalyzed reactions are highly selective and efficient toward specific substrates under mild conditions, which are involved in almost all biological and metabolic processes, serving as the prime protagonists in the chemistry of living organisms at a molecular level.^[^ ^114^ ^]^ It has been demonstrated that enzymes show varied expression levels in many disease‐associated microenvironments including tumors.^[^ ^115^ ^]^ It is worth noting that TME exhibits excessive enzyme secretion comprising matrix metalloproteinases (MMPs), hyaluronidase (HAdase), *γ*‐glutamyl transpeptidase, and esterase compared with normal tissues.^[^ ^116^ ^]^ For example, proteases are able to degrade proteins or peptide substrates. Oxidoreductases could catalyze the electron transfer from the reductant to the oxidant. Kinases mediate the activities of proteins through the phosphorylation process, whereas the opposite action, dephosphorylation, is adjusted by phosphatases. It has been demonstrated that the expression level of MMP‐2 in breast cancer cells MDA‐MB‐231 was about sixfold higher than that in normal mammary cells HS578Bst.^[^ ^117^ ^]^ Additionally, the expression level of HAse in high‐grade bladder cancer was about eightfold higher than that in normal bladder tissue, while the expression level of HAse showed no significant difference between low‐grade bladder cancer and normal bladder tissue.^[^ ^118^ ^]^ Therefore, enzyme‐responsive nanomedicine has become the research hotspot in recent years and served as a promising tool to enhance the cancer immunotherapy.

### 2.6 High‐Level ATP

ATP is a key metabolite that plays a vital role in diverse physiological and pathological processes in vivo.^[^ ^119^ ^]^ It has been shown that the concentration of ATP varies between normal cells and tumor cells, between the extracellular and intracellular environment, and from one organelle to another, which has motivated the design of ATP‐triggered and ATP‐fueled nanomedicine.^[^ ^120^ ^]^ In comparison with ATP levels in normal cells (2537 × 10^−6^ ± 1217 × 10^−6^ m), cancer cells exhibited a 1.2‐fold higher ATP level (3134 × 10^−6^ ± 2135 × 10^−6^ m) due to their faster metabolic processes, excess glycolysis, and fast proliferation and growth.^[^ ^121^ ^]^ It should be noted that mitochondria exhibited the high ATP level of 8.0 × 10^−3^ ± 2.6 × 10^−3^ m while the ATP level in cytoplasm was measured to be 3.3 × 10^−3^ ± 0.5 × 10^−3^ m in liver cells.^[^ ^122^ ^]^ Therefore, the application of ATP as a trigger or a chemical fuel is of high relevance to developing responsive nanomedicine for cancer immunotherapy.

## 3 TME‐Responsive Nanomedicine for Immunotherapy

### 3.1 pH‐Responsive Nanomedicine for Immunotherapy

#### 3.1.1 pH‐Responsive Nanoparticle for Immunotherapy Based on Acid‐Labile Bondsa

**Figure 1:**
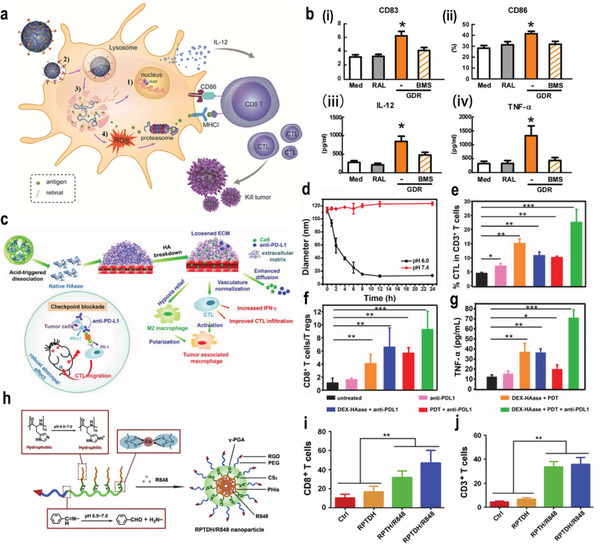
a) Scheme of GDR and GDR/OVA nanovaccine. b) The expression of i) CD83 and ii) CD86 on bonemarrow‐derived dendritic cells (BMDCs) were measured using flow cytometry. The productions of iii) IL‐12 and iv) TNF‐*α* in culture supernatants were measured using enzyme linked immunosorbent assay (ELISA). Reproduced with permission.^[^ ^23^ ^]^ Copyright 2016, Elsevier Ltd. c) The proposed mechanism of enhance PDT and antitumor immune responses induced by DEX–HAase adjuvant and PD‐L1 checkpoint blockade. d) The size change curve of DEX–HAase nanoparticle in pH 6.0 and 7.4 PBS solution. e) CTL infiltration in tumors. CD3^+^CD8^+^ cells were defined as CTLs. f) The ratio of CD8^+^ T cells to regulatory T cells of mice post various treatments. g) The production of TNF‐*α* in serum of mice determined on the ninth day post various treatments. Reproduced with permission.^[^ ^24^ ^]^ Copyright 2019, Wiley‐VCH. h) Schematic illustrations for preparation of RPTDH/R848 nanoparticles. i,j) CD3^+^ (i) and CD8^+^ (j) T cell infiltration in tumor. Reproduced with permission.^[^ ^27^ ^]^ Copyright 2019, Elsevier Ltd. *p*‐Values: *p < 0.05; **p < 0.01; ***p < 0.001.

In recent years, cleavage of chemical bonds has been extensively designed to take advantage of specific stimuli, such as low pH in the TME.^[^ ^123^ ^]^ Drug release mechanisms are divided into direct dissociation of drug molecules from nanomedicines, or disassembly of nanostructures with a size transition from large to small. The most commonly used pH sensitive chemical bonds include hydrazone, imine, oxime, cis‐maleic monoamides, polyacetal and polyketone, ether (usually viny ethers), and ortho ester.^[^ ^124^ ^]^ For example, Ma and co‐workers reported a pH‐responsive nanogel named as galactosyl dextran‐retinal (GDR), which was composed of dextran (DEX) and all‐trans retina with a connection by hydrazone bond (**Figure** **1**a). It was found that ovalbumin (OVA) antigen could be loaded into GDR effectively to form a GDR/OVA nanovaccine. In the tumor acidic environment, the hydrazone bond is cleaved and vitamin A in the GDR/OVA is released and oxidized into the active metabolite all‐trans retinoic acid, which interacts with the nuclear receptor retinoic acid receptor to achieve the enhanced antitumor effect. Furthermore, GDR/OVA nanovaccine triggered lysosomal rupture to increase the intracellular content of ROS and proteasome activation. The in vivo results showed that GDR/OVA can significantly increase the levels of cluster of differentiation (CD) 83 and CD86, which effectively induce the production of mature dendritic cell (DC) marker cytokines interleukin‐12 (IL‐12) and tumor necrosis factor *α* (TNF‐*α*), as shown in Figure 1b.^[^ ^23^ ^]^ Liu and co‐workers reported a type of pH‐responsive liposome composed with dextran and HAdase, which was connected with amide bonds. And, chlorin e6 (Ce6) and programmed cell death‐1 (PD‐L1) were encapsulated into the liposome to achieve the combined effect of photodynamic therapy (PDT) and immunotherapy (Figure 1c). In the slightly acidic TME, the acid‐sensitive amide bonds were rapidly hydrolyzed, which resulted in the dissociation of the nanostructure and controlled release of HAdase (Figure 1d). It was revealed that HAdase can degrade the tumor extracellular matrix (TEM), which relieved hypoxia and enhanced the accumulation of nanomedicine in tumor tissue. In vivo experiments showed that nanomedicine could significantly improve the tumor infiltration of cytotoxic T lymphocytes (CTL), and enhance the CTL/Treg ratio and serum TNF‐*α* level, leading to the superior antitumor effect of PDT–immunotherapy (Figure 1e–g).^[^ ^24^ ^]^ Moreover, Xing and co‐workers designed a type of pH‐responsive vesicle with clinically approved polycaprolactone (PCL)‐*b*‐polyethylene glycol (PEG) and hydrazone bonds, which was encapsulated with endogenous tumor antigen Heat Shock Protein 70 (HSP70)‐chaperoned polypeptides (HCPs) and an oligodeoxynucleotide (ODN)‐based immune adjuvant. It was found that the developed nanomedicine can effectively activate antigen‐presenting cells (APCs), and then triggered the activation of CTL, resulting in a long‐term memory immune response and improved cancer immunotherapy.^[^ ^25^ ^]^

Reshaping the tumor immune microenvironment by regulating the polarization of TAM is a promising immunotherapeutic strategy.^[^ ^125^ ^]^ However, the high interstitial fluid pressure and dense extracellular matrix make it difficult for nanomedicine to reach the tumor site. To solve this dilemma, Jiang and co‐workers developed a dandelion‐like tailorable nanomedicine for TME modulation. Dendrigraft poly‐l‐lysines (DGLs) which can induce tumor autophagy were cross‐linked via a mild‐acid‐responsive linker. A long blood circulation and enhanced tumor penetration were observed by retaining the DGLs in a neutral pH while releasing them in the slightly acidic tumor environment. In vivo experiments demonstrated enhanced macrophage regulation, tumor autophagy, and antitumor efficacy.^[^ ^26^ ^]^ Amino acids are biocompatible compounds that have been widely used in tumor immunotherapy. For example, Yang and co‐workers used glutamic acid and histidine to design a tumor‐targeting and pH‐sensitive polymeric copper chelator for the targeted delivery of resiquimod (R848), a Toll‐like receptor (TLR) 7/8 agonist R848 (Figure 1h). Ingeniously, the benzoic–imine bond in the nanomedicine was cleaved at low pH, leading to the hydrophilic–hydrophobic transitions of poly‐histidine. Therefore, the structure of the nanomedicine was gradually degraded, which could release the R848 to activate the maturation of DCs through the TLR 7‐/8‐mediated signaling pathway (Figure 1i,j). More importantly, copper‐containing polymers can further exert antiangiogenesis and antitumor activities through copper chelation, leading to a synergistic antitumor effect.^[^ ^27^ ^]^

#### 3.1.2 pH‐Responsive Nanoparticles for Immunotherapy Based on Protonation

**Figure 2:**
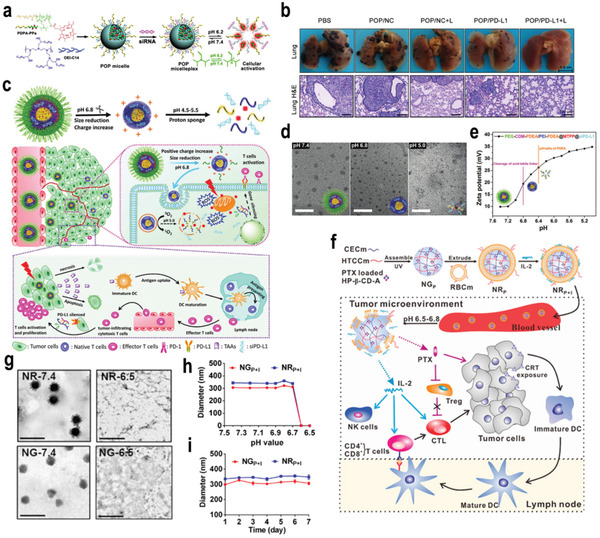
a) Chemical structure of the acid‐activatable POP micelleplexes coloaded with PPa and siRNA. (b) Photographs and H&E staining of the metastatic foci of the B16‐F10 tumors (scale bar 100 µm, *n* = 6). Reproduced with permission.^[^ ^28^ ^]^ Copyright 2016, American Chemical Society. c) Illustration of pH‐response dissociable micelleplex‐mediated photodynamic tumor immunotherapy in vivo. d) TEM images of PCPP@MTPP@siPD‐L1 micelleplexes after various treatments with pH 7.4, pH 6.8, and pH 5.0 for 4 h. MTPP: (5‐(3‐Hydroxy‐p‐(4‐trimethylammonium) butoxyphenyl)‐10, 15, 20 triphenylporphyrin chlorine. e) Zeta potentials variation of PCPP@MTPP@siPD‐L1 micelleplexes under various pH value conditions. Reproduced with permission.^[^ ^29^ ^]^ Copyright 2018, Wiley‐VCH. f) Preparation of NR_P+I_ and schematic illustration of chemo‐immunotherapy. g) TEM images of NG_P+I_ in pH 7.4, NG_P+I_ in pH 6.5, NR_P+I_ in pH 7.4, and NR_P+I_ in pH 6.5. The scale bar is 1 µm. h) pH‐dependent particle sizes of NG_P+I_ and NR_P+I_. i) In vitro stability of NG_P+I_ and NR_P+I_ in saline at 37 °C for 1 week. Reproduced with permission.^[^ ^30^ ^]^ Copyright 2017, American Chemical Society. *p*‐Values: *p < 0.05; **p < 0.01; ***p < 0.001.

Protonation is also one of the most commonly used mechanisms for constructing pH‐responsive nanoplatforms. In this strategy, at physiological pH (7.35–7.45), nanomedicine is deprotonated or deionized, while in the acidic TME (6.5–6.8), the nanomedicine is protonated, leading to charge reversal, structural transformation, or disassembly and the release of the encapsulated drugs.^[^ ^126^ ^]^ In addition, protonation induced by an acidic pH leads to the hydrophilic–hydrophobic phase transition of the nanomedicine, resulting in the aggregation or precipitation of the structure. Meanwhile, charge reversal of anionic polymers caused by the protonation endows the nanomedicine with a positive charge, which could promote enhanced cellular uptake and deeper tumor penetration by electrostatic absorption.^[^ ^127^ ^]^ For example, Li and co‐workers designed a series of acid‐responsive nanoplatforms based on the protonation strategy for tumor immunotherapy (**Figure** **2**a). In their design, cationic micelles, photosensitizers (PS) of pheophorbide A (PPa) and small interfering ribonucleic acid (siRNA) were integrated to form a multifunctional micelle. Due to the aggregation of PS, the fluorescence of the micelle was quenched, and the dark toxicity was small. In a weakly acidic microenvironment, the tertiary amine of poly(diisopropanol amino ethyl methacrylate‐co‐hydroxyethyl methacrylate) (PEG‐*b*‐P(DPA‐*co*‐HEA)) was protonated, leading to the dissociation of the micelles and rapid release of siRNA. It was found that the released siRNA specifically silenced the PD‐L1 on tumor cell membrane, leading to the inhibition of the immune tolerance regulated by the PD‐L1 pathway. Under irradiation by a laser, the nanomedicine stimulated adaptive antitumor immunity by generating singlet oxygen and promoting the uptake of tumor‐derived antigens by an APC reaction, leading to the suppression of tumor metastasis (Figure 2b).^[^ ^28^ ^]^ In addition, Cai and co‐workers developed a pH‐responsive nanocarrier loaded with PS PPa and PD‐L1–blockade siRNA for the synergistic integration of PDT and immunotherapy (Figure 2c). The surface of the micelles was wrapped by a long‐circulating PEG shell, which could fall off in a slightly acidic TME due to the incorporated amide bond, leading to a positive surface charge and smaller particle size, which improved cellular uptake and tumor penetration (Figure 2d,e). Furthermore, the micelle was rapidly protonated in the endo‐/lysosome, leading to its disintegration and the release of PD‐L1–blockade siRNA. In vivo experiments illustrated that the released PD‐L1–blockade siRNA could inhibit the expression of PD‐L1 and immune resistance, while the PPa induced the apoptosis of tumor cells under light irradiation to activate the immune response. Therefore, melanoma growth was significantly hindered and the recurrence rate was reduced via inducing systemic antitumor immune responses.^[^ ^29^ ^]^

Nanogels have been demonstrated as an effective and safe drug delivery system for antitumor immunotherapy.^[^ ^128^ ^]^ For example, Zhang and co‐workers designed a biomimetic nanogel with two oppositely charged chitosan derivatives for the combinatorial chemotherapy and immunotherapy to combat tumors (Figure 2f). Dynamic light scattering (DLS) and transmission electron microscopy (TEM) demonstrated that the nanomedicine could achieve the rapid release of paclitaxel (PTX) and interleukin‐2 (IL‐2) under acidic pH through protonation of —COO^−^ into —COOH and amino groups into positively charged —NH_3_^+^ groups. Therefore, the electrostatic interaction in the nanogel network was transformed into electrostatic repulsion in the TME, leading to the disintegration of the nanogel and the release of drugs (Figure 2g,h). The particle size increased slightly at pH 6.8, and it was almost undetectable at pH 6.5. Additionally, the negligible change in size indicated that nanoparticles presented good stability in vitro (Figure 2i).^[^ ^30^ ^]^ However, it was found that the release ratio of PTX from the nanogels reached up to almost 30% within 24 h at pH 7.4 which may induce the toxicity to normal organs. Therefore, it is very important to improve the drug delivery system and reduce the advanced leakage of PTX. In the preparation of the nanogels, PTX was loaded into the polymers in the first step followed by UV cross‐linking and encapsulation by red blood cell (RBC) membranes. In the fabrication process, PTX may diffuse from the inner of the nanogels to the outside or surface, leading to the rapid PTX release at pH 7.4. Therefore, it is essential to determine the distribution of PTX in the nanogels before the application in vivo to prevent its advanced leakage.

To overcome the endosomal trapping and low immunogenicity of tumor antigens, Liang and co‐workers reported a proton‐driven nanotransformer‐based vaccine, which is composed of a polymer–peptide‐conjugate‐based nanotransformer and antigen peptide (AP). At pH 7.4, the polymers with acetal bonds assembled into spherical nanostructures which could undergo dramatic morphological change to micrometer‐sized nanosheets in acidic environment of endosomes. It was found that the generated nanosheets could rupture the endosomal membrane, leading to the direct delivery of AP to cytoplasm. The reassembled nanosheets could improve the tumor immunity by activating specific inflammatory pathways. Combined with anti‐PD‐L1 antibodies, the nanotransformer‐based vaccine (NTV) could result in over 83 days of survival and about half of the mice produced complete tumor regression.^[^ ^129^ ^]^

#### 3.1.3 pH‐Responsive Nanoparticle for Immunotherapy Based on Other Types

**Figure 3:**
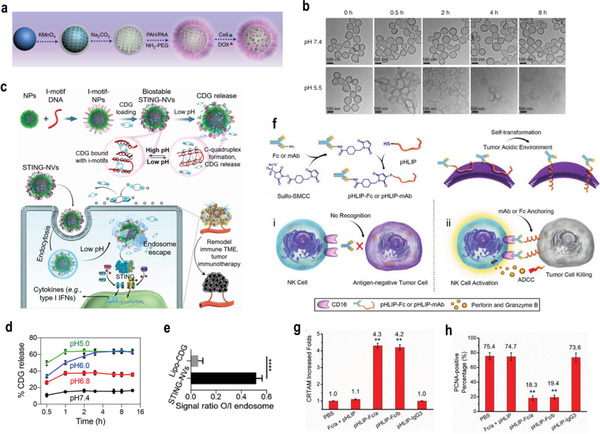
a) A scheme indicating the step‐by‐step synthesis of H‐MnO_2_–PEG nanoparticles and the subsequent dual‐drug loading. b) TEM images of H‐MnO_2_–PEG after incubation in buffers with different pHs (7.4 and 5.5) for various periods of time. Reproduced with permission.^[^ ^32^ ^]^ Copyright 2017, Springer Nature. c) Schematic illustration of pH‐responsive STING‐NVs that efficiently load CDG at physiological pH, stabilized CDG, delivered CDG to immune cells, conditionally released CDG in the acidic endosome, and facilitated endosome escape of CDG for cancer immunotherapy. d) pH‐responsive cumulative CDG release from STING‐NVs. e) The signal ratio of FluoCDG outside/inside (O/I) endolysosome. Reproduced with permission.^[^ ^33^ ^]^ Copyright 2020, Wiley‐VCH. f) The design of pH low insertion peptide (pHLIP)‐modified Fc molecules or antibodies and the proposed immunotherapeutic mechanism. g) Quantification of the increase in NK cell activation (CRTAM‐positive) CRTAM: class‐I restricted T cell‐associated molecule. h) the percentage of proliferating cell nuclear antigen (PCNA)‐positive cells in metastasis tumors treated by various formulations. Reproduced with permission.^[^ ^34^ ^]^ Copyright 2018, Wiley‐VCH. *p*‐Values: *p < 0.05; **p < 0.01; ***p < 0.001.

In addition to the pH‐responsive mechanism mentioned above, there are other types, such as acid‐triggered degradation based on inorganic nanomaterials or conformation transition of proteins. For example, Li et al. prepared a CaCO_3_ nanocarrier encapsulated with the immune stimulants cytosine‐phosphate‐guanosine (CpG) ODNs and indoleamine 2,3‐dioxygenase inhibitor (IDOi). Under acidic conditions in the TME, the CaCO_3_ matrix was disintegrated to release the CpG ODNs, IDOi, and Ca^2+^, which promoted T cell activation and enhanced the cancer immunotherapy.^[^ ^31^ ^]^ Likewise, Liu and co‐workers prepared an acid‐responsive hollow MnO_2_ nanoparticle which was loaded with Ce6 and the chemotherapeutic drug doxorubicin (DOX) (**Figure** **3**a). Under the slightly acidic TME, the MnO_2_ nanoparticle was quickly decomposed, leading to the rapid release of Ce6 and DOX (Figure 3b). Meanwhile, oxygen was also produced in situ, which relieved tumor hypoxia, enhanced the therapeutic effect, and triggered a series of antitumor immune responses. More importantly, in combination with checkpoint blocking therapy, the developed nanomedicine could result in the suppression of distant tumors and tumor metastasis.^[^ ^32^ ^]^

Generally, the conformation of proteins can alter in different pH due to the hydrophilic–hydrophobic changes of the structure. For instance, c‐di‐guanosine monophosphate (CDG) is one of the cyclic dinucleotides (CDNs), which are agonists for the stimulator of interferon gene (STING) and are promising for cancer immunotherapy. However, the therapeutic effect of CDNs is limited by their poor delivery efficiency and biostability. To overcome this limitation, Zhang et al. cleverly designed STING‐activating DNA nanovaccines (STING‐NVs), which could protect CDG from enzymatic degradation and promote the delivery and release of CDG (Figure 3c). Specifically, CDG was loaded into i‐motif DNA nanoparticles via hydrogen bonding between the guanosine (G) in CDG and cytosine (C) in the i‐motif DNA. In the acidic environment, the C in the i‐motif DNA was protonated into C+ and competed with CDG for pairing C:C+ bases, resulting in DNA conformational reconstruction and CDG release (Figure 3d). Additionally, the results of flow cytometry showed that in the STING‐NV group, the ratio of the fluorescence signal intensity of FluoCDG in the tumor cell to the outside of the lysosome was 0.52, which was ≈9 times the ratio of LipoCDG (0.06) (Figure 3e). STING‐NVs promoted the escape of CDG endosomes, which was conducive to the activation of STING. More importantly, STING‐NVs can revert M2‐like macrophages into antitumor M1‐like macrophages, which effectively improved the effect of immunotherapy.^[^ ^33^ ^]^ In addition, Ji et al. reported an antibody or Fc fragment modified with polypeptides to target solid tumors (Figure 3f). Fc fragments or therapeutic monoclonal antibodies were conjugated with low pH‐responsive polypeptides. The conformation of the polypeptides changed from random coil to *α*‐helix in the acidic TME, which can be selectively assembled on the membrane of solid tumor cells, and this led to the effective activation of NK cells and the inhibition of tumor proliferation (Figure 3g,h).^[^ ^34^ ^]^

### 3.2 GSH‐Responsive Nanomedicine for Immunotherapy

**Figure 4:**
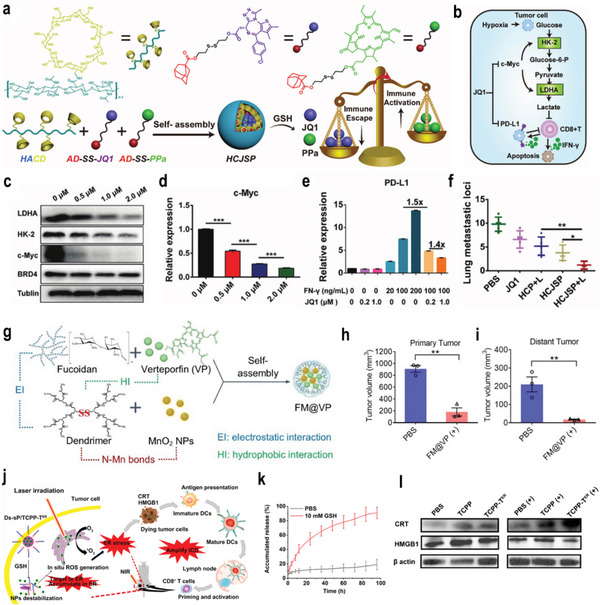
a) Schematic illustration of the HCJSP prodrug nanoparticle prepared via the host–guest interaction between HA–CD and AD‐SS–JQ1 and AD‐SS–PPa. AD‐SS: disulfide bond modified adamantane. b) Schematic illustration of mechanism of combination immunotherapy. c,d) Representative western blot and semiquantitative analysis of the expression of c‐Myc (d), human kidney‐2 (HK‐2), and lactate dehydrogenase A (LDHA) after treated with 0.5, 1.0, or 2.0 µm of JQ1 for 24 h. e) Flow cytometric examination of PD‐L1 expression in Panc02 cells treated with Interferon γ (IFN‐*γ*)/JQ1 alone or combination for 24 h. f) Quantitative analysis of the lung metastatic nodules of in the Pano02 tumor‐bearing mice at the end of the antitumor study. Reproduced under the terms of the CC‐BY license.^[^ ^49^ ^]^ Copyright 2021, Published by Wiley‐VCH. g) FM@VP nanoparticle cluster assembling scheme. h, i) Box plots of tumor volumes in primary (h) and distant tumors (i) on day 29. Reproduced with permission.^[^ ^51^ ^]^ Copyright 2020, Elsevier Ltd. j) Schematic illustration of ER‐targeting PS TCPP‐T^ER^ specifically accumulates in the ER and produces ROS in situ upon laser irradiation to induce ER stress and amplifies ICD. TCPP‐T^ER^: 4,4′,4″,4′″‐(porphyrin‐5,10,15,20‐tetrayl)tetrakis(N‐(2‐((4‐methylphenyl)sulfonamido)‐ethyl)benzamide. k) Reduction‐responsive release of TCPP‐T^ER^ from Ds‐sP/TCPP‐T^ER^ at pH 7.4 with or without 10 × 10^−3^ m GSH. l) Western blot assay of HMGB1 and calreticulin (CRT) expression levels in 4T1 cells treated with Ds‐sP/TCPP or Ds‐sP/TCPP‐T^ER^ with or without 670 nm laser irradiation. Reproduced with permission.^[^ ^52^ ^]^ Copyright 2020, American Chemical Society. *p*‐Values: *p < 0.05; **p < 0.01; ***p < 0.001.

As one of the most widely applied signals in the TME, GSH has been extensively used in the fabrication of responsive nanomedicine for cancer immunotherapy. To fabricate GSH‐responsive nanomedicine, GSH‐cleavable functional groups such as disulfide bonds or diselenide bonds are inserted into the structure of nanomedicine. Some GSH‐responsive prodrugs, such as cis‐platinum, could also be loaded into nanocarriers for immunotherapy. For instance, Li and co‐workers integrated the prodrug cyclodextrin grafted with hyaluronic acid (HA–CD), PPa, and bromodomain‐containing protein 4 inhibitor (JQ1) into a single nanoplatform (**Figure** **4**a). ROS produced by PPa can enhance the immunogenicity of tumor cells and promote the intratumoral infiltration of CTL. JQ1 prodrugs were activated by GSH in the TME, which blocked the transcription of the oncogene c‐Myc, resulting in the inhibition of glycolysis, reducing lactic acid accumulation, and improving the immunosuppressive TME (Figure 4b). Moreover, JQ1 can specifically downregulate the expression of PD‐L1 on the surface of tumor cells to combat the immune tolerance induced by PDT (Figure 4c–f).^[^ ^49^ ^]^ Wang and co‐workers reported a type of liposome loaded with an IDO inhibitor (NLG‐8189), which can be activated by the redox of a phospholipid–porphyrin conjugate. The disulfide bond in the liposome was broken by the high‐level GSH in the TME, leading to the controlled release of NLG‐8189, which reversed the immunosuppressive microenvironment and inhibited the metastasis of cancer.^[^ ^50^ ^]^

The combination of PDT and tumor immunotherapy is a promising antimetastatic tumor method.^[^ ^130^ ^]^ For instance, Chung et al. developed an intelligent nanoplatform based on the functional polysaccharide fucoidan, which consisted of polysaccharide fucoidan and a bioreducible polyamidoamine dendrimer, verteporfin (VP), and MnO_2_ nanoparticles (Figure 4g). In tumor tissue with a high level of GSH, the disulfide bonds in the nanomedicine were broken and disintegrated, thereby releasing the loaded drugs and selectively displaying the fluorescent signal of VP. Besides, VP can inhibit oncogenic signals and weaken tumor‐mediated immunosuppression by inhibiting yes‐related protein. The in vivo results showed that the nanomedicine can not only eradicate primary tumors but also induce an abscopal effects on the distant tumors site (Figure 4h,i).^[^ ^51^ ^]^ In addition, Chen and co‐workers proposed a novel immunogenic cell death (ICD) amplification, an endoplasmic reticulum (ER)‐targeting nanomedicine that can effectively eradicate primary tumors while eradicating distant tumors through abscess effects (Figure 4j). They synthesized GSH‐responsive PEG‐s‐s‐1,2‐distearoyl‐sn‐glycero‐3‐phosphoethanolamine‐N‐[amino‐PEG‐2000] (Ds‐sP) NPs that can load the ER‐targeting TCPP‐T^ER^, which achieved the controlled release of the loaded drugs in the TME (Figure 4k). Ds‐sP/TCPP‐T^ER^ was selectively accumulated in the ER and locally produces ROS, which induced ER stress, amplified the ICD effect, and activated immune cells to enhance the effect of the immunotherapy(Figure 4l).^[^ ^52^ ^]^

Biodegradable mesoporous silicone nanoparticles (MONs) that respond to GSH are the candidate materials for antitumor drug delivery. Disulfide bonds are typically employed as intermediate linkers to fabricate silicon networks. Modified mesoporous silica has the advantages of good biocompatibility, adjustable size, and ability to be loaded with various drugs that could achieve the rapid degradation and controlled drug release. For instance, Xu et al. reported a biodegradable MON loaded with a CpG oligodeoxynucleotide adjuvant and Ce6. The neoantigen peptide was connected to the MONs through disulfide bonds, which can be cleaved in the highly reduced TME, resulting in the rapid lysis of the nanomedicine. Neoantigens can trigger a strong specific CD8*α*+CTL response.^[^ ^53^ ^]^ Moreover, a GSH‐responsive hollow mesoporous silicon nanocarrier has been reported to load hydroxycamptothecin (HCPT) and interfering RNA of monocarboxylic acid transporter 4 (siMCT‐4). Under the action of GSH, siMCT‐4 and HCPT were effectively released, which silenced the expression of MCT, leading to an increase in intracellular lactate and tumor cell apoptosis. In addition, the extracellular lactic acid program reduced the TAM phenotype from M2 the type to M1 type, restored the activity of CD8^+^ T cells, and eliminated the immunosuppressive TME.^[^ ^54^ ^]^ Li and co‐workers fabricated an amphiphilic triblock copolymer PS3D1@DMXAA, which could release the loaded 7‐ethyl‐10‐hydroxycamptothecin (SN38) from the polymer under a high level of GSH in the tumor tissue, which induced tumor cell death and enhanced tumor immunogenicity.^[^ ^55^ ^]^ Tang et al. designed a protein nanogel that could recognize and bind to the surface of T cells, and selectively release protein under the stimulation of high‐level GSH in the tumor tissue, leading to improved antitumor immunotherapy.^[^ ^56^ ^]^

### 3.3 ROS‐Responsive Nanomedicine for Immunotherapy

ROS are crucial signals in the immunosuppressive TME, and they could be used to create ROS‐responsive nanomedicines for cancer immunotherapy.^[^ ^131^ ^]^ Some ROS‐responsive functional groups such as sulfoether groups, peroxalate ester groups, or thioketal groups have been integrated into the structure of nanomedicine, and these groups could be cleaved in the high‐level ROS in TME.^[^ ^132^ ^]^ For example, Yang and co‐workers reported a H_2_O_2_‐sensitive polymer carrier to deliver OVA antigens. The polymer carrier was a three‐armed poly(lactic‐co‐glycolic acid (PLGA) which was coupled to PEG via peroxide ester bonds. Then, the nanomedicine was modified with HA, which can target CD44 cells to promote uptake by immune cells. The obtained nanomedicine achieved the controlled release of antigens by high‐level ROS in the TME, which promoted the maturation of DCs, antigen uptake, and antigen presentation, thereby inducing an effective immune response.^[^ ^63^ ^]^ Furthermore, Yang and co‐workers developed a ROS‐responsive nanomedicine incorporated with a peroxalate ester bond. The peroxalate ester bond was degraded by the high level of H_2_O_2_ in the TME, leading to the controlled release of the loaded antigens to activate immune cells for enhanced cancer immunotherapy.^[^ ^64^ ^]^

**Figure 5:**
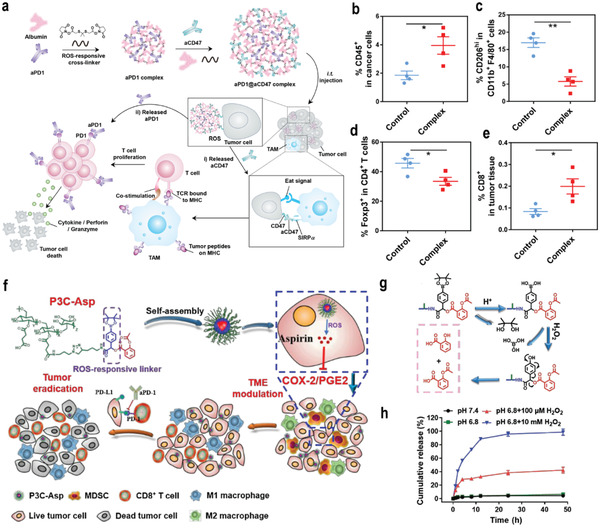
a) Schematic illustration showing the synergistic immunotherapy using the ROS‐sensitive complexes for controlled sequential release of aCD47 and aPD1 in the TME. b–e) Flow cytometry analyzed the percentage of CD45^+^ cells (b), M2‐like macrophages (CD206^hi^F4/80^+^CD11b^+^) (c), CD4^+^Foxp3^+^ T cells (d), and CD8^+^ T cells (e) in B16‐F10 tumors. Reproduced with permission.^[^ ^65^ ^]^ Copyright 2019, American Chemical Society. f) Schematic illustration the mechanism of P3C‐Asp in combination with aPD‐1 in cancer immunotherapy. g)The proposed release mechanism of P3C‐Asp in the presence of H_2_O_2_. h) In vitro aspirin release profiles of P3C‐Asp in phosphate buffer with Tween 80 (0.2%, w/v) at four conditions: pH 7.4, pH 6.8 with 100 × 10^−6^ m H_2_O_2_, and pH 6.8 with 10 × 10^−3^ m H_2_O_2_, *n* = 3. Reproduced with permission.^[^ ^68^ ^]^ Copyright 2019, Chinese Chemical Society. *p*‐Values: *p < 0.05; **p < 0.01; ***p < 0.001.

Despite this great progress, the low response rates and systemic side effects impair further development of immunotherapies in the clinic. Therefore, Gu and co‐workers recently performed a series of innovative studies to design and develop ROS‐responsive nanomedicines to overcome the shortcomings of immune checkpoint inhibitors. For example, a protein complex containing a thioketal linker was developed to achieve the sequential release of anti‐“do not eat me” signal antibodies (aCD47) and aPD1 due to degradation by overexpressed ROS in tumor cells (**Figure** **5**a). Additionally, the nanomedicine could remove the ROS in the TME, which ameliorated the immunosuppressive TME. Moreover, the rapid release of aCD47 activated the innate immune system and enhanced the T cell response, leading to enhanced cancer immunotherapy (Figure 5b–e).^[^ ^65^ ^]^ Gu and co‐workers developed a ROS‐responsive polypeptide gel for the sustained release of aPD‐L1 antibodies and dextro‐1‐methyltryptophan (D‐1MT). Segments of PEG and ROS‐responsive polypeptides were applied to fabricate the gel. It was revealed that the ROS‐responsive gel can not only continuously released aPD‐L1 and D‐1MT but also consumed intracellular ROS, thereby improving the survival rate of T cells and alleviating the immunosuppressive TME.^[^ ^66^ ^]^ Furthermore, they developed another degradable hydrogel, which formed in situ and was loaded with gemcitabine (GEM) and PD‐L1 through poly(vinyl alcohol) (PVA). The gel contained a ROS‐labeled linker, N1‐(4‐boronobenzyl)‐N3‐(4‐boronophenyl)‐N1,N1,N3,N3‐tetramethylpropane‐1,3‐diaminium (TSPBA), which can oxidize and hydrolyze TSPBA in response to ROS, and continuously release GEM and aPD‐L1 to enhance the antitumor response. These results showed that the release rate of GEM and aPD‐L1 from the hydrogel was increased in H_2_O_2_ solution compared with phosphate‐buffered saline (PBS) alone. Most GEM was released within 1 day, whereas aPD‐L1 showed a more sustained release profile, with an 80% releasing ratio within 3 days.^[^ ^67^ ^]^

Combination therapy can make up for the shortcomings of a single treatment method and achieve superior treatment of tumors. Therefore, Chen and co‐workers proposed a straightforward strategy to develop ROS‐sensitive polymer–carboxyl drug conjugates (P3C–Asp), as shown in Figure 5f. P3C–Asp effectively accumulated in tumor tissue and released aspirin or salicylic acid rapidly under the stimulation of overexpressed ROS in the TME (Figure 5g,h). Animal experiments showed that the combination of P3C–Asp with immune checkpoint inhibitors aPD‐1 increased the infiltration of CD8^+^ T cells, M1 macrophages, and the ratio of M1 over M2 macrophages, which effectively inhibited tumor growth without obvious side effects.^[^ ^68^ ^]^ Wan et al. developed a ROS‐responsive nanomedicine modified with HAYPRH (T7) peptide for codelivery of siRNA–PD‐L1 and DOX. Due to the modification of T7, the nanomedicine bound well to the overexpressed transferrin receptor on tumor cells and promoted their uptake by cells. Importantly, siRNA–PD‐L1 can block the inhibitory signal to T cells, stimulate the proliferation of T cells, and enhance the effect of tumor treatment.^[^ ^69^ ^]^

Shape could greatly influence the tumor targeting delivery;^[^ ^133^ ^]^ therefore, combination of shape transformation with ROS sensitive drug delivery could achieve good tumor immunotherapy. Our group constructed self‐delivered nanoparticles by the host–guest interaction between Ce6 conjugated *β*‐cyclodextrin (Ce6–CD) and ferrocene‐modified FFVLG3C–PEG conjugates (Fc–Pep–PEG).^[^ ^134^ ^]^ The nanoparticles, Ce6–CD/Fc–Pep–PEG were 95 nm in physical condition. When they entered the tumor, due to the high level of ROS under laser irradiation, the Fc was oxidized to water‐soluble Fc^+^, and the host–guest interaction was destroyed. Consequently, Fc^+^–Pep–PEG could recombine into nanofibers with higher tumor retention, which continuously catalyze the Fenton reaction to generate •OH and O_2_. As a result, photodynamic and antitumor immune responses were enhanced with good primary tumor and bone metastasis treatment.

### 3.4 Hypoxia‐Responsive Nanomedicine for Immunotherapy

Hypoxia in the TME leads to tumor angiogenesis, growth, and metastasis. Hypoxia can also induce immunosuppression, mainly by upregulating the expression of chemotactic cytokines 22 (CCL 22) and CCL28 and the accumulation of myeloid‐derived suppressor cells and regulatory T cells (Tregs).^[^ ^135^ ^]^ Hypoxia also promotes the conversion of macrophages and neutrophils to the tumor‐promoting M2 phenotype, and inhibits the killing effect of T cells and NK cells.^[^ ^136^ ^]^ In addition, increased levels of anaerobic metabolites, such as adenosine and lactic acid, impair the function of connective tissue growth factors by affecting the production of interferon‐*γ*.^[^ ^137^ ^]^ Furthermore, hypoxia is a cause of treatment resistance, especially for PDT and radiotherapy, where oxygen molecules are essential for the eradication of tumor cells.^[^ ^138^ ^]^ Therefore, designing hypoxia‐responsive nanomaterials would help to achieve enhanced cancer immunotherapy.

**Figure 6:**
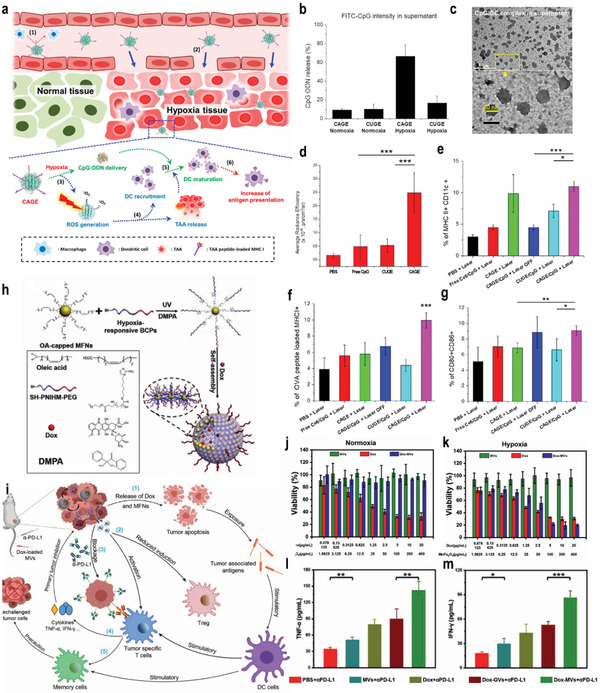
a) A schematic illustration of the preparation and application of the CAGE complex. b) Hypoxia‐responsive CpG ODN release from CAGE. CpG ODN was labeled with fluorescein isothiocyanate (FITC), and the amount of released CpG ODN was investigated by measuring fluorescence intensity of FITC‐labeled CpG in supernatant. c) CpG/glycol chitosan (GC) complex in the supernatant observed by TEM. d) Quantitative assay of CpG ODN accumulated in tumor‐draining lymph nodes (TDLN) after intravenous (i.v.) injection of CAGE/CpG complex. e–g) The corresponding quantification of recruited (e), mature (f), and OVA‐presenting DC population (g). Reproduced with permission.^[^ ^70^ ^]^ Copyright 2018, American Chemical Society. h) Fabrication of DOX–MVs via cooperative assembly of BCP‐grafted MFNs. MVs: manganese ferrite vesicles; MFNs: manganese ferrite nanoparticles. i) Schematic illustration of the mechanism of DOX–MV‐based chemo‐immunotherapy to achieve systemic immune responses. j,k) In vitro cytotoxicity of MVs, DOX, and DOX–MVs against 4T1 cells incubated under normoxic and hypoxic conditions, respectively. l,m) Cytokine levels in the sera from mice isolated 7 days after mice were rechallenged with secondary tumors (on day 47). Reproduced with permission.^[^ ^72^ ^]^ Copyright 2021, Wiley‐VCH. *p*‐Values: *p < 0.05; **p < 0.01; ***p < 0.001.

The depletion of tumor‐infiltrating DC population limits the effectiveness of tumor immunotherapy. Therefore, it is necessary to develop an efficient immune adjuvant delivery system to enhance the activity of DCs. Kim and co‐workers designed a hypoxia‐responsive mesoporous silica nanomaterials (CAGE), which was doped with Ce6 on mesoporous silica (**Figure** **6**a). In addition, PEGylated chitosan was modified onto the surface of the nanomedicine via a hypoxia‐sensitive azobenzene linker. In the tumor hypoxic environment, the azobenzene linker was cleaved, which led to the controlled release of the loaded drugs and maturation of DCs (Figure 6b–g). In vivo experiments indicated that the nanomedicine can significantly inhibit the growth of tumors with the combination of PDT and immunotherapy, leading to the effective inhibition of tumor metastasis.^[^ ^70^ ^]^ Furthermore, Yoon and co‐workers developed a targeted tumor delivery system that was loaded with phthalocyanine derivatives (PcN4) and hypoxia‐sensitive prodrugs banoxantrone (AQ4N). After entering the blood circulation, PcN4 interacted with endogenous albumin dimers and generated supramolecular complexes in vivo, which provided a facile approach for tumor‐targeting PDT and created a more hypoxic TME for the activation of the prodrug AQ4N. In vivo experiments demonstrated that the developed strategy completely removed primary triple‐negative breast cancer (TNBC) and significantly activated CD8^+^ T cells, resulting in a significant antimetastatic effect.^[^ ^71^ ^]^ Recently, Chen and co‐workers developed a hypoxia‐responsive nanovesicle that was composed of manganese ferrite and nitroimidazol‐containing amphiphilic polymers (Figure 6h,i). The developed nanovesicle could rapidly dissociate into individual manganese ferrite particle due to the hydrophilic–hydrophobic transition of the hypoxia‐responsive nitroimidazol group in the TME, leading to the rapid release of DOX and the decomposition of tumor endogenous H_2_O_2_ for tumor hypoxia relief. Therefore, superior antitumor effect was observed in a hypoxic environment by the nanomedicine (Figure 6j,k). In combination with PD‐L1‐mediated checkpoint blockade therapy, the nanomedicine not only achieved a superior effect to suppress the growth of the primary tumor, but also promoted long‐lasting immune memory to inhibit tumor recurrence and metastasis (Figure 6l,m).^[^ ^72^ ^]^

### 3.5 Enzyme‐Responsive Nanomedicine for Immunotherapy

**Figure 7:**
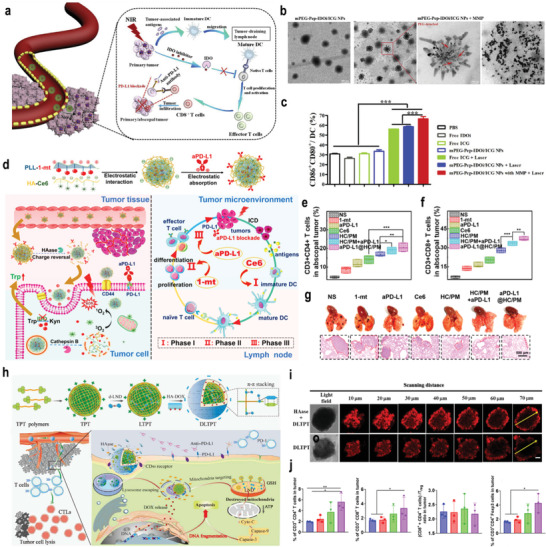
a) Schematic illustration of the TME‐responsive prodrug nanoplatform with deep tumor penetration for efficient synergistic cancer immunotherapy. b) TEM images of mPEG–Pep–IDOi/ICG NPs and mPEG–Pep–IDOi/ICG NPs treated with MMP‐2. mPEG: polyethylene glycol monomethyl ether. c) Expression of the costimulatory molecules CD86 and CD80 on BMDCs induced by different formulations with/without NIR‐laser‐irradiation‐pretreated B16‐F10 cells. NIR: near infrared. Reproduced with permission.^[^ ^74^ ^]^ Copyright 2020, Elsevier Ltd. d) Assembly strategy for aPD‐L1@HC/PM NPs and illustration of the step‐by‐step detached release behavior of aPD‐L1, Ce6, and 1‐mt and the immunotherapy capability via the cascade‐amplifying cancer‐immunity cycle. HC/PM is a mixture of hyaluronic acid (HC) and dextro‐1‐methyl tryptophan (1‐mt)‐conjugated polylysine (PM). e,f) CD3^+^CD4^+^ T cells (e) and CD3^+^CD8^+^ T cells (f) in distant tumors after different treatment. g) Photographs and H&E staining of lung metastatic nodules of the B16‐F10 tumors. Reproduced with permission.^[^ ^77^ ^]^ Copyright 2019, American Chemical Society. h) Schematic diagram of dual‐drug chemo‐ and immune‐combinational therapy mechanism. i) Confocal laser scanning microscope (CLSM) images of DLTPT and HAase+DLTPT incubated with 4T1 multicellular tumor spheroids for 6 h. j) CD4^+^ T cells, CD8^+^ T cells, the ratio of (CD8^+^ T and CD4^+^ T cells) and Treg and CD3^+^CD4^+^ Foxp3 (Treg) in tumor. Reproduced with permission.^[^ ^78^ ^]^ Copyright 2021, American Association for the Advancement of Science. *p*‐Values: *p < 0.05; **p < 0.01; ***p < 0.001.

Tumor deep penetration is important to achieve the optimal antitumor therapeutic index for nanomedicine, and it is especially necessary for large tumors.^[^ ^139^ ^]^ Particle size is an important parameter to determine the penetration ability of a nanomedicine, and small‐sized nanoparticles are more likely to pass through the dense TEM.^[^ ^15^, ^140^ ^]^ In recent years, enzyme‐responsive nanomedicine has offered a solution to solve this problem.^[^ ^141^ ^]^ To improve the tumor penetration of nanomedicine into tumors, Ma and co‐workers developed an enzyme‐responsive prodrug that was composed of a PEGylated IDO inhibitor conjugated by the peptide sequence PVGLIG and indocyanine green (ICG) (**Figure** **7**a). The nanomedicine could transform into small nanoparticles smaller than 40 nm with the degradation by MMP‐2 in the TME, which led to enhanced tumor penetration and cellular uptake (Figure 7b). In vivo experiments showed that the nanomedicine could induce an antitumor immune response, which adjusted IDO‐mediated immunosuppression (Figure 7c). Moreover, it was found that the combination of the developed nanomedicine with PD‐L1 checkpoint blockade synergistically facilitated the antitumor immunity, leading to the inhibition of both primary and abscopal tumors’ growth.^[^ ^74^ ^]^ To reduce the side effects of traditional chemotherapy, Cai and co‐workers fabricated a MMP‐responsive drug delivery system based on mesoporous silica nanoparticles (MSNs) which was immobilized with the substrate peptide PLGLAR through an amidation reaction. In addition, bovine serum albumin was used as the end cap to block the mesopores of polysulfonic mucopolysaccharide. It was found that MSNs responded to the overexpressed MMPs in the TME, which led to the controlled release of the loaded drug and reduced side effects in combination with immunotherapy.^[^ ^75^ ^]^ Amphiphilic triglyceride monostearate (TGMs) are a smart carrier that can specifically respond to MMP‐2 to achieve precise drug release. Wen et al. prepared an enzyme‐responsive nanomedicine (Pd–DOX@TGMs), which loaded DOX and palladium nanoparticles into TGMs. The combination of chemotherapy and photothermal therapy (PTT) promoted the release of dangerous signal molecules, such as high mobility group box 1 protein (HMGB1), calreticulin, and adenosine triphosphate, which improved the immunogenicity of dead tumor cells, and promoted the maturation of DCs and infiltration of T lymphocytes. More importantly, the ICD triggered by the combination therapy enhanced the PD‐L1 checkpoint blocking effect and effectively reversed the immunosuppressive microenvironment.^[^ ^76^ ^]^

In addition, a positive surface charge could enhance the interaction between tumor cells and nanomedicines, which improves tumor cellular uptake and antitumor immunotherapy. Luan and co‐workers developed a three‐in‐one nanomedicine that was composed of Ce6‐conjugated HA, dextro‐1‐methyl tryptophan (1‐mt)‐conjugated polylysine, and aPD‐L1, as shown in Figure 7d. After entering the TME, the developed nanomedicine could be degraded by HAase, which led to the positive charge conversion and enhanced cellular uptake. It was found that the nanomedicine was able to achieve the step‐by‐step detachment of the antigen, leading to sequential antigen presentation, lymphocyte activation, and proliferation/differentiation (Figure 7e,f). In vivo experiments demonstrated that the nanomedicine could effectively inhibit the tumor metastasis, relapse, and postsurgical regrowth due to the cascade‐amplifying cancer‐immunity cycle (Figure 7g).^[^ ^77^ ^]^ Very recently, our group developed a cascade‐targeting, dual drug‐loaded, and core–shell nanomedicine for combinational chemoimmune therapy, as shown in Figure 7h. The developed nanomedicine exhibited long blood circulation and high tumor accumulation due to the negatively charged HA. After entering the tumor tissue, the HA shell on the nanomedicine could be degraded by extracellular overexpressed HAase, leading to decreased size and a positive surface charge, thereby achieving the enhanced tumor penetration and uptake (Figure 7i). In combination with anti‐PD‐L1, the tumor growth was significantly hindered, leading to an intensive immune response against tumor metastasis (Figure 7j).^[^ ^78^ ^]^

Apart from the MMP‐2 and HAase, legumain that could hydrolyze asparagine in proteins and small molecule substrates was also overexpressed in a lot of cancers such as gastric cancer, ovarian cancer, and colorectal cancer. Taking advantage of this feature, our group fabricated legumain‐responsive gold nanoparticles that were loaded with DOX and hydroxychloroquine (HCQ).^[^ ^43^ ^]^ The developed nanomedicine could passively accumulate into the glioma tissue and form in situ aggregates in response to legumain, leading to the enhanced retention of the nanomedicine in tumor. In vivo experiments demonstrated that the combination of DOX with HCQ exhibited promising antiglioma effect. In combination with anti‐PD‐L1 antibody, the nanomedicine was able to neutralize immunosuppressed glioma microenvironment and thus unleash antiglioma immune response, thereby efficiently reducing tumor recurrence.

### 3.6 ATP‐Responsive Nanomedicine for Immunotherapy

**Figure 8:**
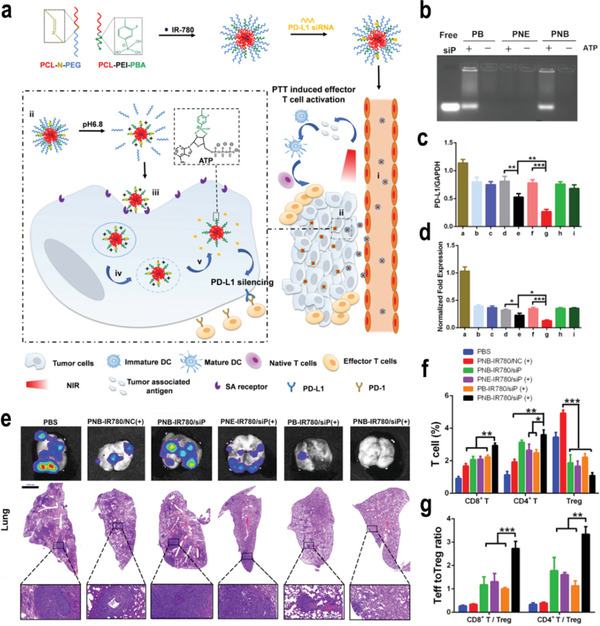
a) Schematic on the preparation process of PNB–IR780/siP and illustration of the pH/ATP cascade‐responsive nanocourier targeting tumor delivery and mediated photothermal tumor immunotherapy in vivo. b) ATP‐triggered uploading of siP from different micelles for 10 min. c) PD‐L1 mRNA expression on 4T1 cells treated with various preparations (*n* = 3, mean ± SD). a: PBS, b: PB–IR780/siP at pH 7.4, c: PB–IR780/siP at pH 6.8, d: PNE–IR780/siP at pH 7.4, e: PNE–IR780/siP at pH 6.8, f: PNB–IR780/siP at pH 7.4, g: PNB–IR780/siP at pH 6.8, h: Lipo–siP at pH 7.4, i: Lipo–siP at pH 6.8. d) Quantitative presentation of PD‐L1 protein. e) Representative bioluminescence images and H&E assays of lungs. f) The frequency of tumor‐infiltrating CD4^+^ T cells, CD8^+^ T cells, and Tregs in mice with various treatments (*n* = 3, mean ± SD). g) CD8^+^ T cells: Treg ratios and CD4^+^ T cells: Treg ratios in the distal tumors (*n* = 3, mean ± SD). SD: standard deviation. Reproduced with permission.^[^ ^82^ ^]^ Copyright 2021, Elsevier Ltd. p‐Values: *p < 0.05; **p < 0.01; ***p < 0.005.

ATP is an important stimulus to improve the specific and controlled release of preloaded drugs from carriers due to its the high expression level in the TME.^[^ ^142^ ^]^ Taking advantage of this, numerous ATP‐responsive nanomedicines have been extensively explored and developed using ATP as a trigger or chemical fuel.^[^ ^119^, ^120^ ^]^ More importantly, ATP‐responsive nanomedicine has been exploited to enhance the antitumor effect of cancer immunotherapy. For instance, He and co‐workers designed a phenylboronic acid (PBA)‐based micelle that encapsulated PD‐L1 siRNA (siP) and infrared dye 780 (IR780). The PEG shell detached from the micelle in the weakly acidic TME, leading to an increased positive charge and PBA exposure for enhanced tumor penetration and uptake (**Figure** **8**a). More importantly, ATP in the TME could bind with PBA, which reduced the positive surface charge of the micelle, leading to the rapid release of the loaded siP and then silence the PD‐L1 (Figure 8b–d). In vivo experiments demonstrated that the combination of PTT with systemic antitumor immune responses triggered by efficient PD‐L1 silencing could not only eliminate 4T1 orthotopic tumor but also inhibit the growth of distant tumors, metastasis, and recurrence due to the generation of immune memory (Figure 8e–g).^[^ ^82^ ^]^ Therefore, ATP‐responsive nanomedicine has become a research hotspot to enhance the antitumor effect of immunotherapy, which may attract more attention in the near future.

### 3.7 Multiple‐Responsive Nanomedicine for Immunotherapy

#### 3.7.1 pH‐ and GSH‐Responsive Nanomedicine for Immunotherapy

**Figure 9:**
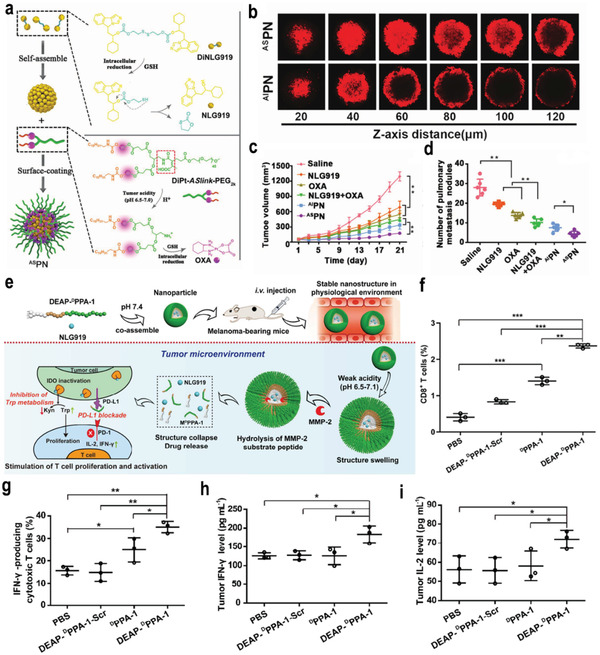
a) Schematic illustration of the BCPN for improved immunotherapy by cooperatively modulating the immune tumor microenvironment. b) Representative CLSM images of fluorescence distribution in 4T1 multicellular spheroid (MCSs) after 12 h incubation with acid‐sensitive binary cooperative prodrug nanoparticle (^AS^PN@NR) pretreated in acid buffers. c) The tumor growth curves in 4T1‐tumor‐bearing mice following the indicated treatments. d) The number of lung metastatic nodules of mice bearing 4T1 tumors at the end of the antitumor study. Reproduced with permission.^[^ ^83^ ^]^ Copyright 2018, Wiley‐VCH. e) The proposed antitumor mechanism of the NLG919@DEAP‐DPPA‐1 nanoparticle. f,g) The percentages of CD8^+^ T cells (f) and IFN‐*γ*‐producing cytotoxic T cells (g) in tumors from various treatment groups (*n* = 3) were analyzed using flow cytometry on day 12 after the commencement of treatment. h,i) The expression of IFN‐*γ* (h) and IL‐2 (i) in tumors from various treatment groups (*n* = 3). Reproduced with permission.^[^ ^85^ ^]^ Copyright 2018, American Chemical Society. p‐Values: *p < 0.05; **p < 0.01; ***p < 0.005.

Because a low pH in the TEM could achieve a surface charge conversion by the protonation strategy and high level of GSH in the tumor cytoplasm could trigger rapid drug release, Li and co‐workers developed a TME‐responsive binary cooperative prodrug nanoparticle (BCPN) that was loaded with the oxaliplatin (OXA) prodrug and NLG919 to improve the effect of cancer immunotherapy by synergistically modulating the immune TME (**Figure** **9**a).^[^ ^83^ ^]^ BCNP exhibited a negative to positive surface charge transition for improved tumor accumulation and deep penetration (Figure 9b). Moreover, OXA and NLG919 achieved the specific activation via a GSH‐mediated reduction reaction. In vivo experiments indicated that GSH‐activated OXA facilitated intratumoral accumulation of CTL by inducing the ICD of tumor cells. NLG919 downregulated IDO‐1‐mediated immunosuppression and inhibited Tregs, leading to the suppression of tumor metastasis (Figure 9c,d). Furthermore, Yang and co‐workers developed a pH‐/GSH‐cascade‐responsive nanomedicine by loading NLG919 and curcumin to overcome the biological barriers and immune resistance.^[^ ^84^ ^]^ The developed nanomedicine achieved the transformable size and a surface charge in response to low pH in the TME. In addition, a high level of GSH in the tumor cytoplasm triggered the rapid release of curcumin and NLG919 for chemotherapy‐improved immunotherapy, which significantly inhibited tumor growth, metastasis, and recurrence in vivo.

#### 3.7.2 pH‐ and Enzyme‐Responsive Nanomedicine for Immunotherapy

Low pH and overexpressed enzymes are two remarkable features in the extracellular TME, which could be used to achieve synergistic controlled drug release in the TEM for immunotherapy. Nie and co‐workers developed an amphiphilic polypeptide self‐assembling nanomedicine for the codelivery of PD‐L1 short D‐peptide antagonist and polyamine 2,3‐dioxygenase inhibitor, which can sequentially respond to pH and enzymes in the TEM (Figure 9e). The hydrophobic domain of the amphiphilic polypeptide was composed of a functional 3‐diethylaminopropyl isothiocyanate (DEAP) and peptide substrate of MMP‐2, whereas the hydrophilic domain was composed of short D‐peptide antagonist of programmed cell death‐ligand 1 (^D^PPA‐1). In the weakly acidic TME, DEAP was protonated, leading to the swelling of the nanomedicine. Moreover, the nanomedicine was completely collapsed due to the cleavage of the peptide substrate by overexpressed MMP‐2 in the TME. The sequential released NLG919 and ^D^PPA‐1 created an environment that was beneficial for the survival and expression of CTL, resulting in the inhibition of melanoma growth and improved overall survival (Figure 9f–i).^[^ ^85^ ^]^ To improve the low response rate to immune‐checkpoint blockade (ICB), a pH and MMP dual‐sensitive micelle was developed that exhibited spatiotemporally controlled release of aPD‐1 and PTX to achieve synergistic cancer chemo‐immunotherapy. Antitumor immunity was triggered by PTX‐induced ICD and the PD‐1/PD‐L1 axis was blocked by aPD‐1 to significantly suppress immune escape, leading to an enhanced therapeutic index.^[^ ^86^ ^]^

#### 3.7.3 GSH‐ and ROS‐Responsive Nanomedicine for Immunotherapy

**Figure 10:**
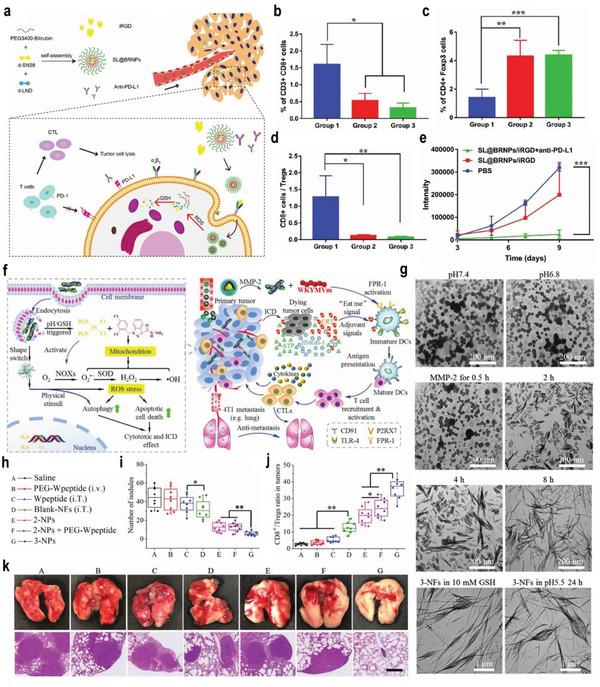
a) The schematic illustration of the construction, drug delivery, and tumor response of SL@BRNPs, and the combinational therapy. b–d) The statistical results of the corresponding immune cells include CD8^+^ T cells (b), Tregs (c), and the ratios of CD8^+^ T cells/Tregs (d). The groups 1, 2, 3, respectively, correspond to group SL@BRNPs/iRGD + anti‐PD‐L1, SL@BRNPs/iRGD, and PBS. iRGD: tumor penetrating peptide (cRGDKGPDC). e) The growth of metastasis semiquantified by bioluminescence intensity. Reproduced with permission.^[^ ^87^ ^]^ Copyright 2019, Wiley‐VCH. f) Schematic illustration of synergistic immunotherapy mechanisms of transformable NPs. g) The TEM images of 2‐NPs incubated in pH 7.4 PBS and in pH 6.8 PBS containing 100 ng mL^−1^ MMP‐2 for 0, 1, 2, 4, and 8 h, and of 2‐NFs (originated from 2‐NPs treated with MMP‐2 for 8 h) in pH 6.8 PBS containing 10 × 10^−3^ m GSH for 12 h as well as in pH 5.5 PBS for 24 h. h) The experimental groups and the meaning of labels. i) The number of nodules on the surface of lungs excised from 4T1‐tumor‐bearing mice at day 26 (*n* = 8). j) CD8^+^/Tregs ratio in tumor tissues (*n* = 8). k) The images and H&E staining of lungs. Mice were treated with saline, PEG–Wpeptide (i.v.), Wpeptide (intratumoral, i.T.), blank NFs (i.v.), 2‐NPs (i.v.), 2‐NPs + PEG–Wpeptide (i.v.), and 3‐NPs (i.v.), respectively (the blue scale bar is 2.5 mm and the black scale bar is 500 µm). Reproduced with permission.^[^ ^146^ ^]^ Copyright 2019, Wiley‐VCH. p‐Values: *p < 0.05; **p < 0.01; ***p < 0.005.

The concentrations of GSH and ROS maintain the balance of the oxidation and reduction states in tumor cells, ensuring the normal function of cell metabolism. Taking advantage of this, our group reported a ROS‐responsive PEGylated bilirubin nanomedicine (BRNP) that was encapsulated with two GSH‐activatable drugs, including dimer‐7‐ethyl‐10‐hydroxycamptothecin (d‐SN38) and dimer‐lonidamine (d‐LND) (**Figure** **10**a). BRNPs could achieve the rapid release of d‐SN38 and d‐LND triggered by ROS after entering the tumor cells. Moreover, d‐SN38 and d‐LND were activated under the stimulation of endogenous GSH, which significantly inhibited the primary breast cancer tumor. Meanwhile, the combination of the nanomedicine and anti‐PD‐L1 antibody hindered the growth of tumors and increased the level of CD8^+^ T cells and ratio of CD8^+^ T cells/T lymphocytes in the tumor, leading to an immune memory effect on inhibiting tumor metastasis (Figure 10b–e).^[^ ^87^ ^]^ In addition, Li and co‐workers developed a GSH and ROS dual‐responsive nanomedicine to achieve the on‐demand delivery and release of the ICD inducer and immune modulators IDOi. After entering the TME, the ROS‐responsive thioketal bonds in the outer amphiphilic block polymer were cleavable by ROS to expose the kernel which could be further broken down by GSH to release IDOi to enhance cancer immunotherapy.^[^ ^57^ ^]^

#### 3.7.4 GSH‐ and Enzyme‐Responsive Nanomedicine for Immunotherapy

Generally, highly effective nanomedicine needs to achieve deep penetration into solid tumors, enhanced cellular uptake, and controlled drug release in tumor cells. To achieve this, Qian and co‐workers designed an easy‐operating procedure to fabricate a therapeutics‐based nanosystem for cancer immunotherapy.^[^ ^143^ ^]^ The developed nanomedicine with a Lyp‐1 sequence could be degraded by overexpressed MMP‐2 in the TME, leading to enhanced tumor penetration and active tumor targeting. Moreover, the high level of GSH in the tumor cytosol led to the rapid release of the loaded drugs, resulting in PD‐1‐/PD‐L1‐blockading‐mediated immunomodulation to enhance tumor immunotherapy. An in vivo experiment demonstrated that the obtained nanomedicine significantly inhibited the primary tumor growth with a combination of PTT, which may provide a classical example to design a simple but powerful nanomedicine for cancer immunotherapy.

As one of the most common primary brain tumor, glioblastoma remains incurable due to the limited accumulation of drugs and the unsatisfactory therapeutic index.^[^ ^144^ ^]^ Therefore, He and co‐workers developed a nanomedicine that respond to GSH and caspase‐3 to control the release of DOX and IDOi for chemo‐immunotherapy. In the TME, high levels of GSH degraded the disulfide bond in the nanoparticle to release DOX. In addition, active caspase‐3 in the TME cleaved the KDEVD peptide segment to release IDOi to reverse the immunosuppressed state for enhanced immunotherapy.^[^ ^145^ ^]^

#### 3.7.5 Triple‐Responsive Nanomedicine for Immunotherapy

In addition to dual‐responsive nanomedicine, triple‐responsive nanomedicine for cancer immunotherapy has been developed in recent years. For example, Zhang and co‐workers designed a triple‐responsive nanomedicine that responded to MMP‐2, pH, and GSH sequentially for synergistic immunotherapy of TNBC, as shown in Figure 10f.^[^ ^146^ ^]^ The developed nanomedicine could achieve the on‐demand structural transformation for optimal size and shape changes, efficient drug delivery, and controlled drug release (Figure 10g). Furthermore, the nanomedicine synergistically amplified the ROS cascade and increased the formation of H_2_O_2_ and toxic •OH, which induced the ICD response and promoted anti‐TNBC immunity by strengthening the interactions between DCs and dying cancer cells (Figure 10h–j). Therefore, the strategy proposed by Zhang and co‐workers achieved significant primary tumor regression and pulmonary metastasis inhibition, which may pave the way for innate and adaptive anti‐TNBC immunity (Figure 10k).

In addition, previous studies have demonstrated that chemo‐immunotherapy could trigger a robust T cell antitumor immune response by inducing ICD.^[^ ^147^ ^]^ However, the therapeutic efficiency of current chemo‐immunotherapies is limited due to inferior drug delivery efficacy and immunosuppression effects in the TME. Li and co‐workers developed a TME‐activatable prodrug vesicle by encapsulating the OXA prodrug and PEGylated PS into a single nanomedicine for cancer chemo‐immunotherapy.^[^ ^148^ ^]^ The developed nanomedicine responded to a weak acid, MMP‐2, and GSH, leading to tumor‐specific accumulation, activation, and deep penetration. Furthermore, codelivery of the OXA prodrug and PS activated the ICD of the tumor cells by immunogenic cell killing, resulting in the efficient inhibition of both primary and abscopal tumor growth as well as the prevention of tumor metastasis and recurrence. Although multiresponsive nanomedicines can improve the accuracy of drug release and enhance therapeutic efficiency, their complex design and preparation processes may impair the potential for clinical applications. Therefore, simple nanomedicines should be pursued to facilitate the development of cancer immunotherapy.

## 4 Summary and Outlook

Immunotherapy has aroused great interest due to its potential for cancer treatment. Although cancer immunotherapy has made significant progress in the past decade, immune‐related side effects and challenges related to the immunosuppressive TME have hindered its clinical application. Stimulus‐responsive nanomedicine has great potential in improving the efficiency of cancer immunotherapy and minimizing side effects through tumor‐specific accumulation, controllable drug release profiles, and combination therapies by integrating multiple treatment options. This review first introduced the latest developments in TME‐responsive nanomedicine for cancer immunotherapy, focusing on the stimuli in the TME, such as pH, GSH, ROS, hypoxia, enzymes, and ATP. Although extensive progress has been achieved, there remain some crucial limitations and challenges that need to be addressed to realize the therapeutic potential of nanomedicines and improve patient care in the clinic.

### 4.1 Biosafety and Simpleness

The biosafety of nanomedicines is a prerequisite for their biomedical application and clinical transformation. The safety profiles of nanomedicine in combination with immunotherapeutic agents should be systemically explored to identify possible host tissue damage or dysfunction of the immune system in the long term. In the design of TME‐responsive nanomedicine, biocompatible or biomimetic materials, especially FDA‐approved materials, should be particularly considered. Currently, there are mainly Abraxane, Doxil, messenger ribonucleic acid (mRNA) nanovaccines, and a biomaterial‐scaffold‐supported autologous vaccine that have been conducted in clinical trials for cancer immunotherapy.^[^ ^149^ ^]^ The combination of nanomedicines with immunotherapy is in the prosperous development stage with a bright future. However, there are currently no TME‐responsive nanomedicines for immunotherapy in clinical trials. The main constraint may be the high complexity of the TME‐responsive nanomedicines being built, which led to the difficulty to achieve the mass production and batch stability. In addition, the therapeutic results from the commonly used mice models could not reflect the real effects on patients owing to the high heterogeneity of tumors in human beings, which increased the difficulty in clinical transitions. Therefore, it is very important for the researchers to reduce the complexity of the current nanomedicines to make them simpler and more reproducible. Moreover, the use of large animals such as rhesus monkey models should be encouraged in the development of TME‐responsive nanomedicines for immunotherapy.

### 4.2 Tumor Targeting

To achieve the TME response of nanomedicine for enhanced cancer immunotherapy, the nanomedicine needs to be effectively delivered to the tumor tissues. However, the long‐term established EPR effect has become controversial, with large differences between humans and animals or among different patients.^[^ ^150^ ^]^ Few nanomedicines have been successfully delivered to tumor tissues, leading to the limited therapeutic index of nanomedicine in clinical trials.^[^ ^151^ ^]^ Despite extensive endeavors to optimize the properties of nanomedicine, increasing the tumor targeting efficiency of nanomedicine to a clinically applicable level is a challenge, which may be due to the multiple biological barriers including uptake by mononuclear phagocyte systems, nonspecific distribution, hemorheological limitation, intratumoral pressure, dense extracellular matrix, and cell membrane internalization. Strategies that only overcome one of these biological barriers cannot bring significant improvements in tumor targeting efficiency. Therefore, the development of versatile biomaterials that could overcome most biological barriers is in desperate demand despite the great challenges.

### 4.3 Therapeutic Efficacy

First, the material composition and structure, physicochemical parameters such as size, surface charge, shape, and stiffness, and dosing and injection routes of nanomedicine must be adjusted to realize the desired pharmacokinetics, tissue or organ‐targeting delivery, optimal efficacy, and minimal toxicity. Particularly, administration timing and sequence of combinatorial agents need to be carefully explored. Immune cells may be eliminated and inactivated in the TME or lymphoid tissues with incorrect sequences of chemo‐immunotherapies, leading to reduced therapeutic efficacy. The timing of the injection of PTX or cyclophosphamide significantly influenced the induction of antitumor T cell responses by CD47 blockade.^[^ ^152^ ^]^ Furthermore, the combination of immunotherapy with other therapies, such as chemotherapy, radiotherapy, PTT, PDT, or chemodynamic therapy (CDT), could also significantly enhance the therapeutic efficacy and reduce side effects, which may represent an important developmental direction for cancer immunotherapy.

### 4.4 Dynamics of the Immune Microenvironment and Personalized Therapy

As the tumor immune microenvironment is dynamic and varies over time, it is essential to personalize and modulate immunotherapeutic interventions at different stages to achieve the optimized therapeutic efficiency. In addition to utilizing imaging technology to observe and monitor the immune response and therapeutic efficacy, an alternative solution is to integrate diagnostic, theranostic, and prognostic functions into systems to form cancer immunotheranostics. Immunotherapy is difficult to make functional for every patient. Therefore, it is necessary to stratify and differentiate patients according to individual differences for superior therapeutic performance. Furthermore, more efforts should be devoted to exploring and screening endpoint biomarkers, which are of great significance to predict patient response, determine the treatment plan, and optimize personalized combinational approaches. It is expected that the application of TME‐responsive nanomedicines in suitable patients for personalized treatment will promote their application in clinical practice.

In summary, TME‐responsive nanomedicine has demonstrated great potential to overcome challenges associated with cancer immunotherapy. TME‐responsive nanomedicine exhibits the advantages of controllable drug delivery and modular flexibility, which could reduce the off‐target toxicity of immunotherapy and immune‐related adverse events. The combination of nanomedicine with immunotherapy can not only eliminate tumors but also trigger the release of tumor antigens and intracellular danger signals, which can initiate systemic antitumor immune responses, leading to the inhibition of tumor recurrence and metastases. There is no doubt that the combination of immunotherapy with TME‐responsive nanomedicine will stand on the threshold of great advances for cancer immunotherapy in the near future.

## Conflict of Interest

The authors declare no conflict of interest.
